# True Grit in Learning Math: The Math Anxiety-Achievement Link Is Mediated by Math-Specific Grit

**DOI:** 10.3389/fpsyg.2021.645793

**Published:** 2021-04-06

**Authors:** Youqing Yu, Liyun Hua, Xingwang Feng, Yueru Wang, Zongren Yu, Tong Zi, Yajun Zhao, Jingguang Li

**Affiliations:** ^1^Leshan Normal University, Leshan, China; ^2^College of Teacher Education, Dali University, Dali, China; ^3^No. 4 Middle School of Binchuan, Dali, China; ^4^School of Education and Psychology, Southwest Minzu University, Chengdu, China

**Keywords:** grit, math anxiety, math achievement, procrastination, Chinese adolescent

## Abstract

In this study, we tested a possible mechanism of the association between math anxiety and math achievement: the mediating role of math-specific grit (i.e., sustaining effort in the face of adversity when learning math). In Study 1, a sample of 10th grade students (N = 222) completed a battery of personality and attitude questionnaires, and math achievement was indexed by curriculum-based examination scores. Mediation analyses indicated that math-specific grit, but not domain-general grit, mediated the relationship between math anxiety and math achievement. In Study 2, we replicated and extended the above findings with another sample of 11th grade students (N = 465). Mediation analyses indicated that math-specific grit and math-specific procrastination played sequential mediating roles in the relationship between math anxiety and math achievement. That is, individuals with higher math anxiety were less gritty in math learning, possibly further leading them to be more procrastinated in performing math work, which may finally result in worse math achievement. In summary, the current study provides the first evidence that math-specific grit may mediate the relationship between math anxiety and math achievement. Furthermore, it also demonstrated the value of math-specific grit over domain-general grit in predicting math success, which invites a broader investigation on subject-specific grit.

## Introduction

Math anxiety is defined as a feeling of fear, tension, and apprehension about math (Ashcraft, 2002). A stable negative association between math anxiety and math achievement has been found across individuals and countries (Foley et al., 2017). By analyzing 747 effect sizes, a recent meta-analytic study revealed a small-to-moderate negative correlation (r = −0.28) of this association (Barroso et al., 2021). The present study seeks to explore the mechanisms underlying this association, which may help in developing methods to mitigate the negative effect of math anxiety on math achievement.

In general, psychologists have discovered that math anxiety may lead to worse math achievement in two scenarios—when taking math tests and during daily math learning (Ashcraft, 2002). When taking math tests, math anxiety may deplete cognitive resources (e.g., working memory and attention), resulting in worse math performance (Ashcraft and Kirk, 2001). During daily math learning, math anxiety may alter individuals' attitude, motivation, and self-confidence toward learning math (Hembree, 1990), resulting in worse math achievement. In this study, we seek to explore a new mechanism explaining the relationship between math anxiety and math achievement in the daily learning scenario—the mediating role of grit.

Grit is one of the most important predictors of academic success [see Eskreis-Winkler et al. (2016) for a review]. The concept was initially developed by Duckworth et al. (2007). They defined grit as trait-level perseverance (referring to sustaining effort in the face of adversity) and passion (referring to consistency in one's interests over time) for long-term goals. Moreover, recent evidence has demonstrated that grit's primary utility in academic achievement lies in the perseverance facet [but see Xu et al. (2020)]. In one meta-analytic study, researchers found that only the perseverance facet added predictive power to academic achievement when controlling for Big Five conscientiousness (Credé et al., 2017). In another meta-analytic study, researchers found that the perseverance facet shows a much larger contribution to academic achievement than the passion facet (Lam and Zhou, 2019). In addition, two facets of grit should not be aggregated together, given that no evidence supporting for a single-factor construct of grit (Guo et al., 2019). Thus, in this study, we used the perseverance facet only to represent the grit construct.

Specifically, we hypothesized that math-specific grit might play a mediating role in the math anxiety–math achievement relationship. Admittedly, there is a lack of empirical studies on math-specific grit in the extant literature. However, based on existing literature on math anxiety and grit, the hypothesized mediation relationships could be illustrated as followed. First, when facing obstacles to learning math, individuals with higher math anxiety levels may be more prone to give up on math learning (i.e., less gritty). This may occur because these individuals have fewer cognitive resources (Ashcraft and Kirk, 2001) and less self-confidence (Hembree, 1990) in facing math-related obstacles than individuals with less math anxiety. Second, giving up in the face of adversity during math learning (i.e., less gritty) may lead to less study time (Duckworth et al., 2007) and more procrastination (Jin et al., 2019), which finally results in worse math achievement. Third, recent literature has demonstrated that domain-specific grit (e.g., grit specific to school learning) can account for more variance in academic achievement than domain-general grit (e.g., Clark and Malecki, 2019; Schmidt et al., 2019). Therefore, we argued that math-specific grit plays a more critical mediating role in the math anxiety-math achievement relationship than domain-general grit. Finally, given that lack of direct evidence regarding the hypothesized relationships, the nature of our investigation is exploratory.

To test the proposed mediation model describing the relationship between math anxiety, math-specific grit, and math achievement, we conducted the current study with Chinese adolescents. In Study 1, we examined whether math-specific grit mediates the relationship between math anxiety and math achievement in one sample of 10th grade students (N = 222). To further verify the specificity of the proposed mediation model, we ruled out the possibility of an alternative model: math anxiety influences math achievement through domain-general grit. In Study 2, we first replicated the results in Study 1 with another sample of 11th grade students (N = 465), which ensured the replicability of the research findings (Pashler and Harris, 2012). In addition, we further explore how math-specific grit influences math learning in depth. As grit makes students less procrastinated (Jin et al., 2019) and procrastination is negatively correlated with academic achievement (Kim and Seo, 2015), we proposed a sequential mediation relationship between math anxiety, math-specific grit, math-specific procrastination, and math achievement. That is, these variables may sequentially influence each other in the above order.

## Study 1

In Study 1, the first goal was to examine whether math-specific grit is negatively associated with math anxiety and positively associated with math achievement, which has not been tested before. Then, we investigated the mediating role of math-specific grit in the proposed mediation model.

### Material and Methods

#### Participants and Procedure

The participants were 222 high school students in 10th grade recruited from Dali, China. The Medical Ethics Committee of Dali University approved the study. We obtained written consent from all participants and their parents. The mean age of the participants was 16.1 years (SD = 0.5 years); 65.8% of the participants were female. Participants completed a set of paper-based questionnaires in classrooms with their classmates. The main goal of recruiting these participants is to investigate the determinants of academic achievement among adolescents (Li et al., 2020).

#### Measures

Math anxiety was measured by the 9-item Abbreviated Math Anxiety Scale (AMAS) (Hopko et al., 2003). The participants rated the extent of their anxiety when facing nine math learning scenarios with response options ranging from 1 (low anxiety) to 5 (high anxiety). The scale consists of two subscales: learning math anxiety (e.g., “listening to another student explaining a math formula”) and math evaluation anxiety (e.g., “thinking about an upcoming math test 1 day before”). The Chinese version of the AMAS was established through a translation and back-translation process by the authors of the current study. In this study, the Cronbach's αs for the AMAS, learning math anxiety subscale, and math evaluation anxiety subscale were 0.86, 0.72, and 0.85, respectively. To index math anxiety in a simple and comprehensive manner, the total score of the AMAS was used subsequently.

Domain-general grit was measured by the 4-item perseverance of effort subscale of the Grit-S (Duckworth and Quinn, 2009). Sample items include “I finish whatever I begin.” The Chinese version of the Grit-S used in the study has been validated in Chinese adolescents (Li et al., 2018). To measure math-specific grit, we adapted the perseverance of effort subscale of the Grit-S by adjusting the items to math learning context (Schmidt et al., 2019). Sample items include “When learning math, I finish whatever I begin.” The response options for both scales range from 1 (not at all like me) to 5 (very much like me). In this study, the Cronbach's αs for domain-general and math-specific grit were 0.59 and 0.77, respectively.

Math achievement was indexed by the scores on the final term examination for the first semester (full score = 150) of 10th grade, which reflects the students' learning progress in the first half of the 10th grade year. The achievement tests were administered in the same month as the questionnaire session.

#### Data Analysis

Statistical analyses were performed using R 3.4.3 with the “lavaan” R package (Rosseel, 2012). For the mediation analysis, the indirect effect was estimated with the bootstrap method (subsample N = 5000), and standardized regression coefficients were reported. In addition, given that several measures were translated or adapted versions of original scales, we performed confirmatory factor analyses to assess their factor structure. In general, they showed satisfactory structural validity; see Supplementary Materials for details.

### Results and Discussion

Table 1 presents the means, standard deviations, and correlations for the study measures. As expected, math anxiety was negatively associated with math achievement (r = −0.20, p < 0.01). In addition, after controlling for age and gender, the correlations between math anxiety and math achievement remained significant (partial r = −0.16, p = 0.02). Thus, our study replicated the negative link between math anxiety and math achievement reported in the literature.

**Table 1:** Means, standard deviations (SD), and correlations among the major variables in Study 1.

	**Mean**	**SD**	**1**	**2**	**3**	**4**
1. Math anxiety	2.03	0.70	–			
2. Math achievement	94.51	19.10	−0.20^**^	–		
3. Domain-general grit	3.41	0.68	−0.27^**^	0.07	–	
4. Math-specific grit	3.43	0.73	−0.48^**^	0.27^**^	0.49^**^	–

* *p < 0.01*.

Next, we examined the relationship between math-specific grit and math anxiety/achievement, which has not been tested before. As expected, math-specific grit was negatively correlated with math anxiety (r = −0.48, p < 0.01) and positively correlated with math achievement (r = 0.27, p < 0.01).

**Figure 1:**
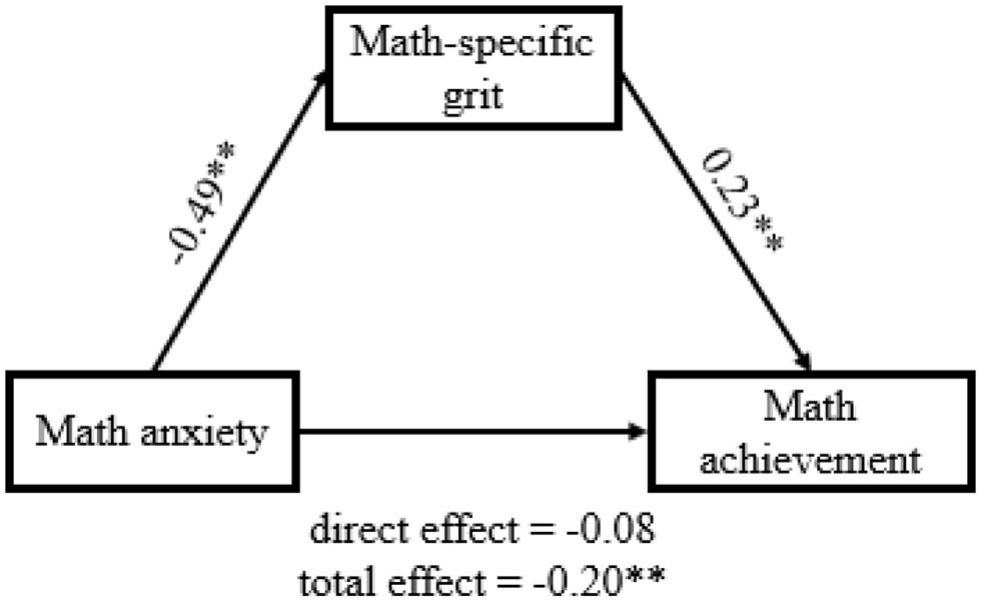
Mediation model of math anxiety as a predictor of math achievement mediated by math-specific grit. Standardized regression coefficients are displayed for all paths. **p < 0.01.

Critically, we tested whether math-specific grit plays a mediating role in the association between math anxiety and math achievement. Mediating analyses confirmed the proposed mediation model. After including math-specific grit as an intermediate variable, the associations between math anxiety and math achievement were reduced from −0.20 (95% CI = [−0.33, −0.06]) to −0.08 (95% CI = [−0.23, 0.07]) (Figure 1) and became statistically non-significant. In addition, the indirect effect (β = −0.11, 95% CI = [−0.19, −0.03]) accounted for 57.9% of the total effect. Therefore, individuals with more math anxiety were prone to being less grittier when learning math, making them obtain worse math examination scores.

Finally, it is unlikely that math anxiety influences math achievement through domain-general grit. As expected, domain-general grit and math-specific grit shared a large amount of variance (r = 0.49, p < 0.01). However, when testing the mediation role of domain-general grit in the math anxiety-achievement link, no significant mediation effect was found (indirect effect = −0.002, 95% CI = [−0.04, 0.04]), which might be caused by the small and insignificant association between domain-general grit and math achievement.

## Study 2

The purposes of Study 2 were twofold. First, to ensure the replicability of the results, we directly replicated the major findings of Study 1 with another independent adolescent sample. Second, we further extend the above findings by testing the proposed sequential mediation model of math anxiety, math-specific grit, math-specific procrastination, and math achievement.

### Material and Methods

#### Participants and Procedure

The participants were 465 high school students in 11th grade recruited from Dali, China. The Medical Ethics Committee of Dali University approved the study. We obtained written consent from all participants and their parents. The mean age of the participants was 17.1 years (SD = 0.54 years); 60.9% of the participants were female. Participants completed a set of paper-based questionnaires in classrooms with their classmates.

#### Measures

We used the same tools from Study 1 to measure math anxiety, domain-general grit, and domain-specific grit, and found similar reliability scores for the AMAS (Cronbach's α = 0.83), perseverance of effort subscale of the Grit-S (Cronbach's α = 0.60), and math-specific grit (Cronbach's α = 0.83) as those in Study 1.

Math achievement was indexed by the scores on the final term examination for the first semester of 11th grade (full score = 150), which reflects the students' learning progress in the first half of the 11th grade year. The achievement tests were administered in the same month as the questionnaire session.

In addition, the short form of the Academic Procrastination Scale (APS-S) was translated (Yockey, 2016). To measure math-specific procrastination, we adapted it by adjusting the items to the math learning context. Sample items include “When learning math, I put off projects until the last minute”. The response options for both scales range from 1 (strongly disagree) to 5 (strongly agree). In this study, the Cronbach's α for the adapted version of the short form APS-S was 0.87.

#### Data Analysis

We used the same statistical procedures and tools as those used in Study 1. Due to school absence, seventeen students did not take part in the math achievement test. Maximum likelihood estimation method was used to handle missing data in the mediation analysis because it generally produces unbiased estimates in various situations (Baraldi and Enders, 2010).

### Results and Discussion

Table 2 presents the means, standard deviations, and correlations for the study measures. In Study 2, we replicated the main correlation patterns between math anxiety, math achievement, domain-general grit, and math-specific grit. In addition, as expected, math-specific procrastination was negatively correlated with math-specific grit and math achievement.

**Table 2:** Means, standard deviations (SD), and correlations among the major variables in Study 1.

	**Mean**	**SD**	**1**	**2**	**3**	**4**	**5**
1. Math anxiety	2.14	0.71	–				
2. Math achievement	82.62	20.40	−0.32^**^	–			
3. Domain-general grit	3.06	0.73	−0.28^**^	0.03	–		
4. Math-specific grit	3.09	0.84	−0.45^**^	0.33^**^	0.52^**^	–	
5. Math-specific	2.27	0.93	0.39^**^	−0.36^**^	−0.36^**^	−0.55^**^	–
procrastination							

** *p < 0.01*.

Next, we replicated the findings of the mediation relationship in Study 1. After including math-specific grit as an intermediate variable, the associations between math anxiety and math achievement were significantly changed from −0.32 (95% CI = [−0.42, −0.22]) to −0.23 (95% CI = [−0.33, −0.12]). In addition, the indirect effect (β = −0.10, 95% CI = [−0.15, −0.05]) accounted for 31.3% of the variance in the total effect. However, no significant mediation effect was observed when the mediator was replaced by domain-general grit (β = 0.02, 95% CI = [−0.01, 0.05]). Thus, the mediation results observed in Study 1 were successfully replicated in Study 2.

**Figure 2:**
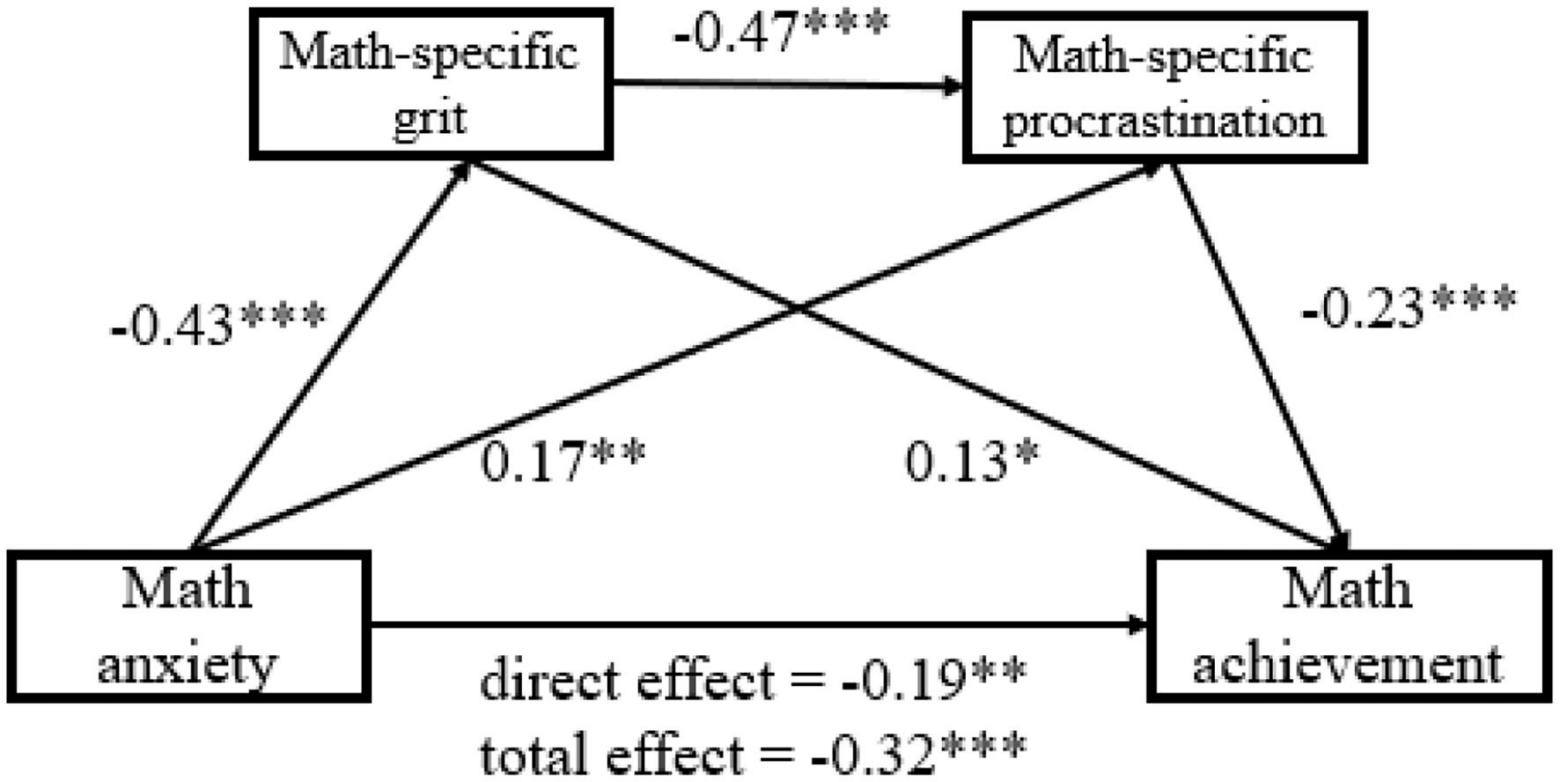
Sequential mediation model of math anxiety as a predictor of math achievement mediated by math-specific grit and math-specific procrastination. Standardized regression coefficients are displayed for all paths. ***p < 0.001; **p < 0.01; *p < 0.05.

Finally, we tested how math-specific grit and math-specific procrastination play multiple mediating roles in the association between math anxiety and math achievement (Figure 2). First, the indirect effects via math-specific grit alone (β = −0.05, 95% CI = [−0.11, −0.008], 38.7% of the overall indirect effect), and math-specific procrastination alone (β = −0.04, 95% CI = [−0.08, −0.01], 27.7% of the overall indirect effect) were both significant, suggesting that both mediators could independently mediate the relationship between math anxiety and math achievement. Second, and more importantly, the mediation pathway of math anxiety → math-specific grit → math-specific procrastination → math achievement was also significant (β = −0.05, 95% CI = [−0.08, −0.02], 33.6% of the overall indirect effect), supporting a sequential mediation model. That is, math anxiety may first decrease math-specific grit, which in turn increases math-specific procrastination and finally results in a lower level of math achievement. Finally, the overall indirect effect (β = −0.14, 95% CI = [−0.20, −0.08]) accounted for 42.4% of the variance in the total effect (β = −0.32, 95% CI = [−0.43, −0.22]), supporting a partial mediation relationship.

## General Discussion

In this investigation, we aimed to explore how math anxiety is linked to math achievement by testing the mediating role of math-specific grit. In both Study 1 and Study 2, we found replicable mediation relationships. That is, math-specific grit, but not domain-general grit, mediates the association between math anxiety and math achievement. Moreover, in Study 2, we extend the meditation model found in Study 1 by adding the effect of math-specific procrastination. Our findings support the proposed sequential mediation model with math-specific grit and math-specific procrastination as sequential mediators of the relationship between math anxiety and math achievement.

The current study contributed to the literature in two novel aspects. First, we provide a new explanation of the well-known relationship between math anxiety and math achievement—the mediating role of math-specific grit. That is, math anxiety might make students less persistent in learning math, such as being more procrastinated, doing less math work, and shrinking back when facing difficulties, which might result in worse math grades. These findings may shed light on mitigating the negative effect of math anxiety on math achievement. Educators need to develop methods to break the vicious chain of math anxiety, math-specific grit, math-specific procrastination, and math achievement. For example, educators may develop training programs to increase math-specific grit among individuals with intense math anxiety, such as altering their mindset about learning math (Bettinger et al., 2018; Wang et al., 2018; Xu et al., in press), which may further help to improve their math achievement.

Second, we demonstrated the predictive power of subject-specific grit over domain-general grit when predicting math achievement. In Duckworth and her colleague's original conceptualization of grit, they emphasized that grit reflects individual tendencies in “a variety of domains (e.g., not just work or school) (pp. 1089)”. However, recent studies have supported the existence and power of domain-specific grit. Some researchers have developed several domain-specific grit scales, such as the Academic Grit Scale (Clark and Malecki, 2019) and English as a Foreign Language Grit Scale (Ebadi et al., 2018). In addition, some researchers have adapted the original Grit scale to specific domains, including sports and school (Cormier et al., 2019; Schmidt et al., 2019). Consistent with other studies on domain-specific grit, our study demonstrated the predictive power of domain-specific grit over domain-general grit when predicting outcomes in the corresponding domain. However, to the best of our knowledge, the current study provides the first evidence in the math domain. These results implied that math educators should pay more attention to students' grit toward learning math than their domain-general grit.

Several limitations of this research should be addressed by future explorations. First, given the cross-sectional nature of the current study design, we did not provide causal evidence supporting the relationship between the variables. Therefore, experimental or longitudinal designs are needed to explore the causal direction of the mediation model. Second, the internal consistency for the domain-general grit is not high, which might be caused by the limited number of items used (i.e., four items). Thus, future studies may replicate the current findings with improved design of psychometric properties. Third, our sample included only Chinese high school students, which constrains the generalization ability of the research findings. Therefore, replicating the findings in other school grades (e.g., primary school and junior middle school) and different ethnicities (e.g., Caucasian population) is needed.

In conclusion, this study provides a novel explanation of the relationship between math anxiety and math achievement by revealing the mediating role of math-specific grit. Future studies are needed to explore how the mediation model found in this study is distinct and connected to known models for the relationship between math anxiety and math achievement (Hembree, 1990; Ashcraft and Kirk, 2001). In addition, researchers and educators are needed to develop methods to mitigate the negative effect of math anxiety on math-specific grit, which may help students with high math anxiety gain better achievement in math.

## Data Availability Statement

The raw data supporting the conclusions of this article will be made available by the authors, without undue reservation.

## Ethics Statement

The studies involving human participants were reviewed and approved by the Medical Ethics Committee of Dali University. Written informed consent to participate in this study was provided by the participants' legal guardian/next of kin.

## Author Contributions

JL conceived and designed the study. YY, LH, XF, YW, ZY, and TZ contributed to data collection. YY, YZ, and JL analyzed data and wrote the paper. All authors reviewed and approved the manuscript.

## Conflict of Interest

The authors declare that the research was conducted in the absence of any commercial or financial relationships that could be construed as a potential conflict of interest.
