# Supplementary material for: True Grit in Learning Math: The Math Anxiety-Achievement Link Is Mediated by Math-Specific Grit
Source: Front Psychol. 2021 Apr 6;12:645793. doi: 10.3389/fpsyg.2021.645793 (PMC8055855; doi:10.3389/fpsyg.2021.645793)
Supplement: Supplementary file 1 [file Table_1.docx]

Supplementary Material

# Supplementary Tables

**Table S1. Fit indices in confirmatory factor analyses for translated and adapted scales**

| Scales | Models | χ^2^(df) | CFI | RMSEA | SRMR |
| --- | --- | --- | --- | --- | --- |
| Study 1 |  |  |  |  |  |
| AMAS | Two-factor | 56.80 (26) | 0.96 | 0.07 | 0.05 |
| Math-specific grit | Single-factor | 17.10 (2) | 0.95 | 0.19 | 0.06 |
| Study 2 |  |  |  |  |  |
| AMAS | Two-factor | 106.46 (26) | 0.94 | 0.08 | 0.05 |
| Math-specific grit | Single-factor | 4.84 (2) | 0.99 | 0.06 | 0.02 |
| Math-specific procrastination | Single-factor | 36.66 (5) | 0.97 | 0.12 | 0.03 |

Notes. AMAS = Abbreviated Math Anxiety Scale; CFI = Comparative Fit Index (> .95 for good fitting), RMSEA = Root Mean Square Error of Approximation (< .08 for good fitting), SRMR = Standardized Root Mean Square Residual (< .08 for good fitting).

# Supplementary Figures


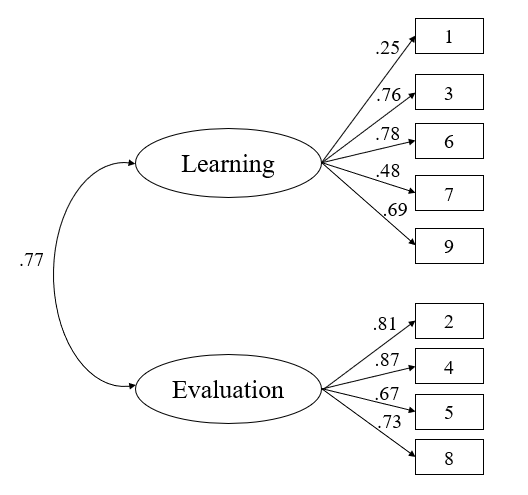


**Figure S1 Standardized factor loadings for the two-factor model of the abbreviated math anxiety scale (Study 1)**


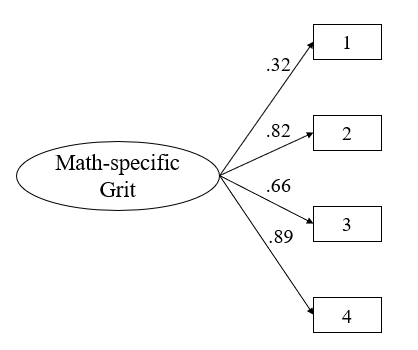


**Figure S2 Standardized factor loadings for the single-factor model of the math-specific grit scale (Study 1)**


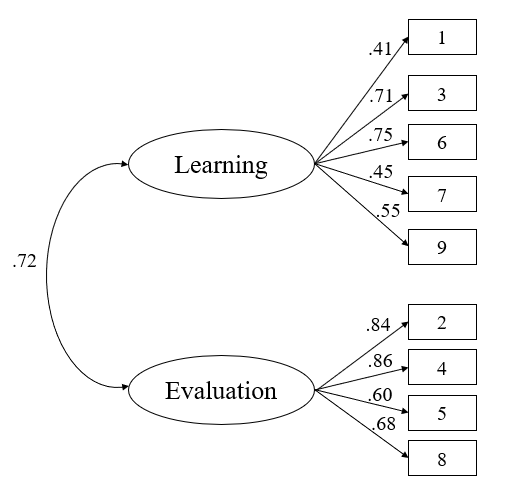


**Figure S3 Standardized factor loadings for the two-factor model of the abbreviated math anxiety scale (Study 2)**


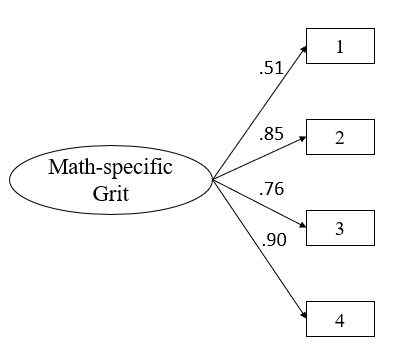


**Figure S4 Standardized factor loadings for the single-factor model of the math-specific grit scale (Study 2)**


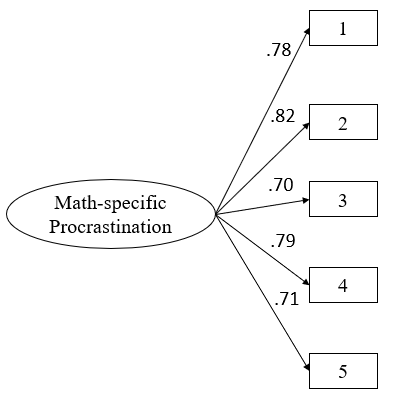


**Figure S5 Standardized factor loadings for the single-factor model of the math-specific procrastination scale (Study 2)**
